# Comparison of Species Composition and Floristic Components of Soil Sediment Spore pollen in the Ailaoshan Mountain Forest of China

**DOI:** 10.1038/s41598-019-48324-9

**Published:** 2019-08-19

**Authors:** Su-Nan He, Cheng-Yuan Hao, Wei Zhao

**Affiliations:** School of Surveying and Land Information Engineering, Henan Polytechnic University, Jiaozuo City, 454000 China

**Keywords:** Environmental impact, Tropical ecology

## Abstract

As the natural boundary between the Yunnan-Guizhou Plateau and the Hengduan Mountains in China, the large-scale prominent terrain of Ailaoshan Mountain leads to obvious regional differences in climate and vegetation. In this article, a comparative analysis is made on both species composition and floristic components of spore pollen (SP) in two sample soil plots near the main peak of Ailaoshan Mountain. The results show that there is a remarkable difference between the western and eastern sample plots. The climate or meteorology characteristics are likely the main reasons accounting for the differences in habitat characteristics and plant sample investigation results. The tropical genera elements of sediment SPs are higher in the western sample plot (6.8%) than in the eastern sample plot (4.7%) because the western plot is controlled by the Indian summer monsoon at all times. The north temperate elements of sediment SP are lower in the western plot (13.4%) than in the eastern plot (26.4%) because the eastern plot is evidently sometimes influenced by the East Asian winter monsoon. In general, Ailaoshan Mountain may be one of important physical geographical boundaries between the interacting regions influenced by both the Indian summer monsoon and the East Asian winter monsoon.

## Introduction

Vegetation is the result of the comprehensive effects of environmental factors such as water, climate, soil, topography and other factors on different scales. Since the early 20^th^ century, the correlation between the species composition of vegetation and its environmental ecological factors has become one of the key research directions in ecological science^1^. However, due mainly to the increasing disturbance by human activities, the habitat, composition, structure and function of primary vegetation have changed, which has resulted in the distortion of their corresponding relationships. Therefore, primary habitat and environmental information can no longer be correctly obtained according to modern vegetation conditions^2^. How to acquire the species compositions of the original vegetation is one of the important issues in modern vegetation restoration. SP is preserved in the stratum for a long period of time with features such as high yield, small individual size, resistance to acids or alkalis, and resistance to high temperature. Thus, SP has already become an important source of information for restoring species composition, structural composition, systematic function, and habitat status of primary vegetation^3–5^.

SPs include flowering plant pollen and cryptophyte spores. The former are mainly male germ cells of angiosperms and gymnosperms, while the latter are germ cells of cryptic plants that develop directly or indirectly into new individuals after detachment from their parents. While SPs are not exactly the same in function, they can effectively reflect the compositions of vegetation species and infer the primary habitat. According to the spatial location, SPs can be divided into three categories, i.e., air SPs, surface SPs and sediment SPs^6^. The air SPs have low research value due to the extreme instability of their spatial positions, while the surface SPs also have low research value due to their close relationships with modern vegetation, who can be seen or obtained for everyone. As an indicator of past habitat conditions, sediment SPs and their combinations can have important implications for the determination of sedimentary age, regional environmental change, inference of human ecological processes, and the correlation between plant communities and their habitat factors^7–9^.

Recently, researchers have conducted extensive and in-depth scientific research on SP as a medium, involving the representativeness of SP, the correlations between SP and modern vegetation, and the restoration of primary habitat by using the correlations among SP, vegetation and environment^10–13^. With the improvement of segregation, identification, statistical techniques, and improvement in the integration of numerical analysis methods, SP combinations have been used to study the correlations between vegetation components and ecological factors^14,15^. That is, research on SP-vegetation-environment correlations is already a hot topic. To date, a large number of scientific studies have been conducted on the correlations between SP and vegetation, while studies on soil sediment SPs in forests are rare^16^. This present study did not follow the routine application of SP analysis. The relationship between plant communities and habitats was also not explored, nor were the age of deposition or the history of environmental change determined. Human ecological processes were also not explored; however, the species composition and floristic components of SPs in soil sediments in an alpine forest are compared between two sample plots beside a high mountain known as Ailaoshan Mountain. The conclusions of this study could be used to restore or reflect the ecological effects of topography, climate and other environmental factors on primary vegetation.

## Results

### Comparison of SP species composition

Based on the data in Table 1, a total of 1015 woody pollen, 70 herb pollen, and 553 fern spores were identified in the western plot. Among the woody pollens, Lithocarpus, Castanopsis, Phyllanthus, Pinus and Alnus were ranked the top five, with the percentages being 28.2%, 17.5%, 13.4%, 12.7%, and 10.9%, respectively. Together, these pollens accounted for 82.7% of the total woody pollen, and their respective proportions are all higher than 10%, but the sixth proportion is only 2.9%. Among the herbaceous pollens, the numbers of Araliaceae and Artemisia were the two highest, accounting for a total of 65.7%. The fern spores appeared in large numbers and were dominated by Polypodium, Davallia and Lepisorus, which together accounted for 92.2%. Of these spores, Polypodium accounted for 69.6% of the total fern spores.

**Table 1:** Composition comparison of soil sediment SPs between the western and eastern plots.

SP	Western plot			Eastern plot		
	Genus (family)	quantity	Proportion/%	Genus (family)	quantity	Proportion/%
Woody pollens	22 (17)	1 015		24 (20)	1 155	
	Lithocarpus	286	28.2	Castanopsis	340	29.4
	Castanopsis	178	17.5	Pinus	288	24.9
	Phyllanthus	136	13.4	Alnus	185	16.0
	Pinus	129	12.7	Lithocarpus	169	14.6
	Alnus	111	10.9	*Quercus*(D)	30	2.6
	Hamamelidaceae	29	2.9	Phyllanthus	26	2.3
	*Quercus*(D)	27	2.7	Hornbeam	21	1.8
	Myrica	26	2.6	*Quercus*(E)	20	1.7
	*Quercus*(E)	25	2.5	Elacocarpaceae	20	1.7
	Ericaceae	13	1.3	Theaceae	13	1.1
Herb pollens	(8)	70		(7)	48	
	Araliaceae	28	40.0	Artemisia	16	33.3
	Artemisia	18	25.7	Araliaceae	12	25.0
Fern spores	(5)	553		(6)	104	
	Polypodium	385	69.6	Davallia	46	44.2
	Davallia	63	11.4	Polypodium	33	31.7
	Lepisorus	62	11.2	Selaginellacea	12	11.5
Sum	/	1 866	/	/	1 356	/

In the eastern plot, a total of 1155 woody pollens, 48 herb pollens and 107 fern spores were identified. Among the woody pollens, Castanopsis, Pinus, Alnus and Lithocarpus were ranked the top four, with percentages of 29.4%, 24.9%, 16.0% and 14.6%, respectively, which accounted for 84.9% of the total pollens, and their respective proportions are all higher than 10%, but the fifth proportion is simply 2.6%. Herbaceous pollens were dominated by Artemisia and Araliaceae, which accounted for 58.3% of the total. The fern spores were fewer, and three dominant genera included Drynaria, Polypodium and Selaginellacea, which accounted for 87.4% of the total.

In terms of the quantity of SPs, the woody pollens and herbaceous pollens in these two plots were roughly the same, but the fern spores differed considerably, and the western plot was more than five times that of the eastern plot. There were 228 SPs from the western plot and 49 from the eastern plot that were not identified but were included, the total amount of SPs in the western plot was 37.6% higher than that in the eastern plot, which indicates that the original habitat of the western plot is more resistant to external disturbances and richer in biomass to a certain extent. In terms of the species classification of the identified SPs, woody pollens, herbaceous pollens, and fern spores in the western plot, there were 17 families, 8 families, and 5 families, respectively, and in the eastern plot, there were from 20 families, 7 families, and 6 families, respectively. This species composition of SPs indicates that the western and eastern plots are very similar in terms of species diversities of primary vegetation. In addition, for the woody pollens, the predominant families of the two sample plots are Fagaceae, including Castanopsis, Lithocarpus and Quercus, and both Pinaceae families, including the Pinus and Betulaceae families, which includes Alnus, accounted for a large proportion. The herbaceous pollens were dominated by Araliaceae and Artemisia families. However, there were some obvious differences. For instance, as is known that the Quercus woody can be classified as deciduous and evergreen, and the ratio of evergreen to deciduous pollens in the western plot was 25:27, while that in the eastern plot was 20:30. The ratio of evergreen components in the western plot was significantly higher than that in the eastern plot, which reflected the adequacy of precipitation and solar energy in the western plot and the superiority of the hydrothermal combination. The Ailaoshan Mountain interception of the warm and humid air currents from the Bay of Bengal may be the main reason, which has resulted in abundant water and heat and their good combination in the western plot, but the opposite case in the eastern plot.

### Comparison of SP floristic components

In general, flora is a general term for all types of vegetation in a certain region and is a natural part of plant species. Research on the plants of contemporary flora in China has mainly focused on the analysis of the geographical components of all plants in a natural geographical area and ultimately determined the sources of these species, but there have been few reports on the causes of vegetation pattern formation. Many studies have shown that the flora in the Yunnan Province belongs to the East Asia flora originating from tropical or subtropical regions, while the flora in this study area belongs to the old-world tropical flora in tropical Asia, which is divided into western and eastern subregions by Ailaoshan Mountain as the boundary^17,18^. The research results of Zhu showed that with increasing latitude, the world’s widespread species and temperate flora components in the adjacent area of Ailaoshan Mountain are increasing, while the components of tropical flora show a decreasing trend^19–21^. However, the vegetation changes in floristic composition caused by nonlatitude factors and genetic analyses need to be strengthened^22^; this research addresses this issue.

According to the classification system of Wu *et al*.^23^, SPs in the eastern and western plots of Ailaoshan Mountain belong to 9 types of flora (Table 2). In terms of quantity, these SPs are dominated by components of the North Temperate and South Temperate Discontinuous Zones, which are greater than 60% whether in the eastern plot or western plot, while the components of both the World Widespread and the North Temperate Zones also account for a rather large proportion. These figures also reflect the cross-transitional nature of this study area at the intersection and demarcation of the Pan-Arctic plant flora and the paleotropical plant flora. Because of the uncertainty in the source of SPs, the flora with a small number of SPs in this study were not analysed here, including three flora zones, such as the Discontinuous Zone of East Asia and North America, the Temperate Intermittent Zone of Europe-Asia and South America, and the Old-world Tropical Zone in Table 2.

**Table 2:** Flora comparison of soil sediment SPs between the western and eastern plots.

Flora zones	Western plot		Eastern plot	
	Quantity	Proportion/%	Quantity	Proportion/%
North Temperate and South Temperate Discontinuous Zones	661	60.9	756	62.8
World Widespread Zone	204	18.8	70	5.8
North Temperate Zone	145	13.4	318	26.4
East Asia and Tropical South America Discontinuous Zones	38	3.5	37	3.1
Pantropical Zone	23	2.1	15	1.2
Tropical Asia to Tropical Africa Zones	13	1.2	4	0.3
East Asia and North America Discontinuous Zones	1	0.1	1	0.1
Temperate Intermittent Zones of Europe-Asia and South America	0	0.0	1	0.1
Old-world Tropical Zone	0	0.0	1	0.1
Sum	1 085	100	1 203	100

The following three points must be emphasized. First, SPs of the World Widespread Zone account for 18.8% of the total quantities in the western plot and only 5.8% in the eastern plot, which indicates that the natural ecological conditions of the western plot are relatively superior to those of the eastern plot, i.e., the western plot is more suitable for the survival of universal plants. Second, the amounts in SPs of the North Temperate Zone in the western and eastern plots varied widely (145:318) and accounted for 13.4% and 26.4% of the total SPs, respectively, which indicated that the eastern plot was significantly affected by the East Asia winter monsoon, while the western plot was less affected. Third, the tropical components of the SPs in the western plot, including the Discontinuous Zone of East Asia and tropical South America, the Pantropical Zone, and the Tropical Asia to Tropical Africa Zones, are larger than those in the eastern plot, which were 6.8% to 4.7%, respectively. Therefore, the western plot was more affected by the Indian summer monsoon than the eastern plot. Based on the above analysis, the tropical components in the western plot are relatively high, probably due to the control of the hot-humid airflow of the Indian summer monsoon from the southwest. However, the SP flora in the eastern plot may be not controlled by this hot and humid vapour system. The main reason for the differences in SP flora compositions of two sampling plots should be the barrier effect of the great Ailaoshan Mountain on atmospheric movement, i.e., Ailaoshan Mountain should be the boundary between the Indian summer monsoon and the East Asia winter monsoon in the study area.

## Discussion

This comparative research on the species compositions and floral elements of the SPs in mountainous soil sediment are not conventional contents of modern SP analysis^24,25^, so there have been few related studies except for the following studies. Based on the SP data of different soil profile sediments at both Gaishan Mountain and Pingshan Mountain in Fuzhou of the Fujian Province, China, Hao *et al*. concluded that there were different characteristics of SP assemblages in different mountain locations and different soil depths^26^. That is, the location of the mountain and the soil depth can be indicated to a certain extent by the SP assemblage means of the soil sediments. Additionally, Chang *et al*. and Li *et al*. showed that the SP in the soil sediments in the subtropical evergreen broad-leaved forests was dominated by local SPs as a supplement to foreign SPs because the latter can only be transported by passing through fluid materials, such as the atmosphere and rivers^27,28^. The results of these studies are the theoretical basis of this study. The results of these studies indicate that the effect of some systemic habitat factors on the vegetation components can be re-inferred through the assemblage characteristics of mountainous soil SP. To compare the influence degree of the relevant research areas, the humid region exerted more influence than the arid region, while the high-cover area was stronger than the low-cover area^29,30^. In short, this comparative research on the species compositions and floral elements of the SPs in the subtropical evergreen broad-leaved forest soil can reflect the differences in the environmental characteristics of native vegetation to a certain extent.

The research team’s previous study on the effects of large-scale barriers at Ailaoshan Mountain has been fruitful, and some of the conclusions are similar to the results of this study, or which can partly explain the results of this study. First, in the comparison of regional climate complexity measures, we used a complex parameter, known as permutation entropy, for time series based on a comparison of neighbouring values and explained the dual effects of the great terrain and atmospheric circulation on the regional climate. Both the temperature permutation entropy and the precipitation permutation entropy displayed a decreasing trend from west to east^31^. Second, in relation to modern vegetation and ecological factors, compared with those in the eastern region, the average annual precipitation and annual average solar radiation on the western side of Ailaoshan Mountain are relatively high, and both water and heat are sufficient, and the combination is good. Therefore, the coniferous forests are broad-leaved evergreen plants, which are high in composition^32^. Third, in the comparison of the beginning of the rainy season, the stations on the eastern side of Ailaoshan Mountain in 2001 entered into the rainy season mostly before the fifth five-day of April, while the stations on the west side were mostly in the first or second five-day of May. In other words, the trapping effect of the large mountain body of Ailaoshan Mountain on the strong, hot and humid marine monsoon airflow is one of the main reasons accounting for the disparity in the beginning of the rainy season between the western and eastern stations^33^, basically bounded by the main peak of Ailaoshan Mountain. Finally, based on basic data such as topography, climate, and vegetation, the mathematical model of self-organizing feature mapping, known as SOFM, was applied to study the ecogeographic demarcation of the area adjacent to Ailaoshan Mountain. The results showed that the great Ailaoshan Mountain not only blocked the southwest summer monsoon from the Indian Ocean, but the mountain is also a barrier for the southwest mountainous region in China from the northern Asia cold dry air^34^. These four conclusions agree with the differentiation in the species composition and floristic components of SP between the eastern and western sample plots in this study.

## Conclusions

In the present study, the SPs in soil sediments of the subtropical evergreen broad-leaved forests were used as media. The composition of plant species and floristic components between the western and eastern plots of the location near the main peak of Ailaoshan Mountain were compared and analysed. The following four main conclusions were obtained. First, on the west side of Ailaoshan Mountain, the main habitat features of the western plot were humid, fog, and massive bryophytes attached to the surface or trunks. The numbers or types of arbours, shrubs, grasses, and ferns were greater than those in the eastern plot. Differences in climate or meteorological characteristics may be one of the main reasons accounting for the differences in habitat features and plant sample investigation results. Second, the differences in the species composition between the eastern and western plots indicated hydrothermal heterogeneity. Compared with that in the eastern plot, the total amount of SPs in the western plot was 37.6% more than that of the ferns and mosses, which comprised the main differences. The evergreen and deciduous components of the Quercus plant in the SPs were approximately the same as those in the western plot, but the evergreen composition was lower in the eastern plot. Furthermore, there were significant differences in the floral component of the SPs in these two sampling plots. Relatively speaking, the higher component of the Tropical Zone in the western plot indicated that the southwest Indian summer monsoon had a stronger affect, while the SPs in the eastern pot had a higher composition of the North Temperate Zone, which indicated that the East Asian winter winds from the north had a significant impact. Finally, the barrier function of Ailaoshan Mountain was the main cause of the large differences in SP characteristics between the two plots. This barrier function mainly manifested itself in the interception of water vapor and air barrier. In this sense, Ailaoshan Mountain should be one of the regional boundaries under the joint action of both the Indian summer monsoon and the East Asian winter monsoon.

## Methods

### Study site

The elevation of Ailaoshan Mountain is more than 2 000 metres above sea level. The mountain belongs to the north vein of the south branch of Yunling Mountain and is oriented in the northwest-southeast direction. The mountain comprises a combination of three natural geographical units of the Qinghai-Tibet Plateau, the Hengduanshan Mountains, and the Yunnan-Guizhou Plateau in China. The main peak is considered to be the boundary. The western and eastern sides are not only different in terms of climatic characteristics but also distinct in vegetation differentiation. In terms of climate, the study area has an obvious dry season with the replacement characteristics of the rainy season in one year. The November to April dry season is dominated by the plateau winter monsoon and the south branch westerly winds, and the weather is relatively dry. The May to October rainy season is significantly affected by two water vapour sources: one is from the Bengal Bay and belongs to the Indian Ocean while the other source is from the South China Sea and belongs to the Pacific Ocean, and the weather is relatively hot and humid. In terms of vegetation, the study area is the largest evergreen broad-leaved forest in the tropical-subtropical regions of China. The study area is not only located at the demarcation zone of the Pan-Arctic and paleoplants but is also at the intersection of various geographical flora components.

In this study, we mainly compared the species compositions and faunal compositions of SP in soil sediments on the west and east sides of the main peak of Ailaoshan Mountain (Daxueguoshan Mount, 3166 m above sea level) at the junction of Xinping County and Zhenyuan County. For comparison, two 10 m × 10 m sampling plots are selected within the zonal vegetation type, i.e., subtropical evergreen broad-leaved forest. Ecogeographical features are similar: sunny slopes, large forest openings, and vegetation coverage of approximately 60%. The soil type is dark brown soil without exposed bedrock and with weak erosion. The sample survey results of the eastern and western plots of Ailaoshan Mountain are as follows. The western plot is located at 24°03′35.0″N, 101°23′57.3″E, and 2 525 m above sea level within Zhenyuan County. The local microclimate characteristics are cold, humid and foggy, and the prominent ecological feature is a large number of mosses. First, the arbour trees in this sample plot include 7 Anneslea fragrans from family Theaceae, 5 Osmanthus fragrans from family Oleaceae, 5 Machilus yunnanensis and 4 Lindera communis from family Lauraceae, and one Castanopsis orthacantha from family Fagaceae. In general, there are a total of 22 plants. Second, there are more shrub plants, mainly including Litsea pungens, Arrow bamboo, Camellia tsaii, Illicium majus, and so on. Third, the herbs are widely grown and mainly include Tupistra fimbriata from the family Liliaceae, Tetrastigma napaulense from the family Vitaceae, and so on. Lastly, the most prominent feature of this sample plot is the characteristics of numerous fern plants, including Asplenium, Sword Ferns, Sweet Fern and others. The eastern plot is located at 24°19′30.3″N, 101°19′01.1″E, and 2 234 m above sea level, within Xinping County. The local microclimate is characterized by low humidity without fog. The prominent ecological feature is the litter layer under the forest in this sample plot. First, there are 15 Schima superba in the family Theaceae, 4 Alnus nepalensis trees in the family Betulaceae, one Castanopsis fargesii in family Fagaceae, which is a total of 20 tree types. Second, the shrub layer is dominated by Radix Berberidis with an average plant height of 1.5 m. Third, the herb layer is dominated by Chromolaena Odorata in the family Asteraceae and a large number of Phyllagathis cavaleriei and Hispid Arthraxon. However, there are few fern plants. In contrast, relative to those of the eastern plot, the habitat of the western plot is mainly characterized by more moisture or fog, so a large number of mosses are attached to the surface or trunks. Although the number and types of trees in the west plot are 22 and 5, and those in the east plot are 20 and 3, the difference is rather small. However, there are many shrubs, herbs and ferns that flourish in the western plot. Table 3 shows the multiyear average numerical comparison of climatic factors. The amount of precipitation in the west plot is greater than that in the east plot during both the rainy season and the dry season, and annual precipitation is 487 mm higher. The annual total solar radiation is larger in the west plot than in the east plot by 221.3 MJ/m^2^, where the dry season radiation is 195.8 MJ/m^2^ higher than that during the rainy season. However, in terms of the extreme temperatures, temperature extremes are greater in the east plot than in the west plot. If the altitude factor influence on temperature is considered, the extreme temperatures of the two sites are basically the same because the altitude of the western plot is 291 m higher than that of the east plot. To a great degree, this difference in climatic factors may be one of the main reasons accounting for the differences in habitat characteristics and vegetation sample survey results.

**Table 3:** Comparison of the main climatic factors between the western and eastern plots.

Plots	Precipitation/mm		Temperature/°C		Radiation/MJ m^−2^	
	Rainy season	Dry season	High extreme	Low extreme	Rainy season	Dry season
Western	1 279.4	247.4	21.3	2.1	3 103.7	3 041.0
Eastern	884.0	155.8	23.5	3.8	3 078.2	2 845.2

### Soil sampling

The field sampling timings in this study were as follows: field sampling on the eastern plot and western plot were conducted on November 22 and November 24, 2014, respectively. Only one soil profile was collected for each plot. Each soil cross-section profile was approximately 40–50 cm in depth, and the soil sample was collected every 10 cm, i.e., four soil samples were collected for each cross-section profile. Each soil sample was evenly mixed, and 10 g was taken for subsequent laboratory measurements.

### SPs segregation

10–15% hydrochloric acid was added to disperse the soil cement to remove calcium carbonate, and the grass roots, fine leaves and other debris were removed by sieving through a 40-mesh screen. Fresh water was added, and samples were washed after 12 h, then the water was changed at least six times. Next, 40% hydrofluoric acid was added so as to remove minerals, and the samples were rinsed with water. Finally, the sample solution was sieved through a 7 μm diameter sieve cloth for approximately 3 minutes in an ultrasonic cleaning dish.

### SPs identification

All SPs were identified by Mr. Luo Yunli, a major professional operator in this study and a research associate from the State Key Laboratory of Systematic and Evolutionary Botany in Institute of Botany, Chinese Academy of Sciences.

### SPs acquirement

All SP assemblages identified from the 4 samples in each plot were used as data of each plot for laboratory analysis.

## References

[1] Liu XP (2012). Changes in vegetation-environment relationships over long-term natural restoration process in Middle Taihang Mountain of North China. Ecol. Eng..

[2] Li MY, Xu QH, Zhang SR, Li YC (2015). Indicator pollen taxa of human-induced and natural vegetation in Northern China. Holocene.

[3] Urrego LE, Gonzalez C, Uran G, Vorenberg JP (2010). Modern pollen rain in mangroves from San Andres Island, Colombian Caribbean. Rev Palaeobot. Palyno..

[4] Felde VA, Peglar SM, Bjune AE, Grytnes JA, Birks HJB (2014). The relationship between vegetation composition, vegetation zones and modern pollen assemblages in Setesdal, southern Norway. Holocene.

[5] Mander L, Punyasena SW (2014). On the Taxonomic resolution of pollen and spore records of earth’s vegetation. Int. J. Plant Sci..

[6] Luo CX (2015). Comparison of modern pollen distribution between the northern and southern parts of the South China Sea. Int. J. Biometeorol..

[7] Yeloff D, Hunt C (2005). Fluorescence microscopy of pollen and spores: a tool for investigation environmental change. Rev. Palaebot. Palyno..

[8] Wang LC (2014). Late Holocene environmental reconstructions and their implications on flood events, typhoon, and agricultural activities in NE Taiwan. Clim. Past.

[9] Jouffroy-Bapicot I, Vanniere B, Iglesias V, Debret M (2016). 2000 years of grazing history and the making of the Cretan mountain landscape, Greece. PloS One.

[10] Collier KJ, Wright-Stow AE, Smith BJ (2004). Trophic basis of production for mayfly in a North Island, New Zealand, forest stream: contributions of benthic versus hyporheic habitats and implications for restoration. N. Z. J. Mar. Freshwater Res..

[11] Court-Picon M, Buttler A, De-Beaulieu JL (2006). Modern pollen/vegetation/land-use relationships in mountain environments: an example from the Champsaur valley (French Alps). Veg. Hist. Archaeobot..

[12] Lindstrom S, Mcloughlin S (2007). Synchronous palynofloristic extinction and recovery after the end Permian even in the Prince Charles Mountains, Antarctica: implications for palynofloristic turnover across Gondwana. Rev Palaeobot. Palyno..

[13] Pan T, Dai EF, Wu SH (2010). Relationship between surface pollen and modern vegetation in southwestern China. J. Mt. Sci..

[14] Li YC (2009). Modern pollen assemblages of the forest communities and their relationships with vegetation and climate in northern China. J. Geogr. Sci..

[15] Dale MB, Allison L, Dale PER (2010). A model for correlation within clusters and its use in pollen analysis. Community Ecol..

[16] Blyakharchuk T, Eirikh A, Mitrofanova E, Li HC, Kang SC (2017). High resolution palaeoecological records for climatic and environmental changes during the last 1350 years from Manzherok Lake, western foothills of the Altai Mountains, Russia. Quatern. Int..

[17] Li R, Kraft NJB, Yang J, Wang Y (2015). A phylogenetically informed delineation of floristic regions within a biodiversity hotspot in Yunnan, China. Sci. Rep..

[18] Liu SY, Zhu H, Yang J (2017). A phylogenetic perspective on biogeographical divergence of the flora in Yunnan, Southwestern China. Sci. Re..

[19] Zhu H (2015). Biogeography of Shangriive on biogeographical divergen. Phytotaxa.

[20] Zhu H (2016). A biogeographical comparison between Yunnan, Southwest China, and Taiwan, Southeast China, with implications for the evolutionary history of the East Asian flora. Ann. Mo. Bot. Gard..

[21] Zhu H, Chiang TY (2017). Floristic characteristics and affinities in Lao PDR, with a reference to the biogeography of the Indochina peninsula. PloS One.

[22] Cuesta F (2017). Latitudinal and altitudinal patterns of plant community diversity on mountain summits across the tropical Andes. Ecography..

[23] Wu, Z. Y., Sun, H., Zhou, Z. K., Li, D. Z. & Peng, H. *Floristics of seed plants from China*. 28–33 (In Chinese) (Science Press, Beijing, 2010).

[24] Essenberg CJ (2013). Scale-dependent shifts in the species composition of flower visitors with changing floral density. Oecologia.

[25] Gong WC (2015). Floral scent composition predicts bee pollination system in five butterfly bush (Buddleja, Scrophulriaceae) species. Plant Biol..

[26] Hao, Y. L., Xuan, Y., Zhou, Y. Z., Tang, W. J. & Guo, J. Specificity of pollen complex in Fuzhou Mountainous soils. *Forensic Sci*. *Tech*. **41**(1), 35–39 (In Chinese) (2016).

[27] Chang, J., Hui, Z. C., Geng, H. P., Hu, X. F. & Pan, B. T. Modern Pollen Transportation Process in the Middle Reach of the Heihe River. *Scientia Geographica Sinica* **37****(****12****)**, 1925–1932 (In Chinese) (2017).

[28] Li Q (2018). Spatial variability and long-term change in pollen diversity in Nam Co catchment (central Tibetan Plateau): Implications for alpine vegetation restoration from a paleoecological perspective. Sci. China Earth Sci..

[29] Pauling A, Rotach MW, Gehrig R, Clot B (2012). A method to derive vegetation distribution maps for pollen dispersion models using birch as an example. Int. J. Biometeorol..

[30] Kujau A (2013). Reconstructing Valanginian (Early Cretaceous) mid-latitude vegetation and climate dynamics based on spore-pollen assemblages. Rev Palaeobot. Palyno..

[31] Hao, C. Y., Wu, S. H. & Li, S. C. Measurement of climate complexity using permutation entropy. *Geogr*. *Res*. **26****(****1****)**, 46–52 (In Chinese) (2007).

[32] Hao, C. Y., Zhang, Y. L. & Wu, S. H. Comparisons on EVI spatial variation and causes exploration among different mountains in the Southwest Yunnan province of China. *J*. *Mt*. *Sci*. **27****(****1****)**, 14–23, (In Chinese) (2009).

[33] Hao CY, Zhang HB (2014). Study on rainy season onset time the Longitudinal Range-gorge Region and its reasons. J. Sat. Oceanogr. Meteor..

[34] Hao CY, Wang LC (2016). Regional clustering for ecological geographical parameters based on SOFM model. Nat. Environ. Pollut. Tech..

